# A ruthenium dimer complex with a flexible linker slowly threads between DNA bases in two distinct steps

**DOI:** 10.1093/nar/gkv864

**Published:** 2015-10-10

**Authors:** Meriem Bahira, Micah J. McCauley, Ali A. Almaqwashi, Per Lincoln, Fredrik Westerlund, Ioulia Rouzina, Mark C. Williams

**Affiliations:** 1 Department of Physics, Northeastern University, Boston, MA 02115, USA; 2 Department of Chemistry and Chemical Engineering, Chalmers University of Technology, Gothenburg, Sweden; 3 Department of Biology and Biological Engineering, Chalmers University of Technology, Gothenburg, Sweden; 4 Department of Biochemistry, Molecular Biology, and Biophysics, University of Minnesota, Minneapolis, MN 55455, USA

## Abstract

Several multi-component DNA intercalating small molecules have been designed around ruthenium-based intercalating monomers to optimize DNA binding properties for therapeutic use. Here we probe the DNA binding ligand [μ-C4(cpdppz)_2_(phen)_4_Ru_2_]^4+^, which consists of two Ru(phen)_2_dppz^2+^ moieties joined by a flexible linker. To quantify ligand binding, double-stranded DNA is stretched with optical tweezers and exposed to ligand under constant applied force. In contrast to other bis-intercalators, we find that ligand association is described by a two-step process, which consists of fast bimolecular intercalation of the first dppz moiety followed by ∼10-fold slower intercalation of the second dppz moiety. The second step is rate-limited by the requirement for a DNA-ligand conformational change that allows the flexible linker to pass through the DNA duplex. Based on our measured force-dependent binding rates and ligand-induced DNA elongation measurements, we are able to map out the energy landscape and structural dynamics for both ligand binding steps. In addition, we find that at zero force the overall binding process involves fast association (∼10 s), slow dissociation (∼300 s), and very high affinity (*K_d_* ∼10 nM). The methodology developed in this work will be useful for studying the mechanism of DNA binding by other multi-step intercalating ligands and proteins.

## INTRODUCTION

**Figure 1.**
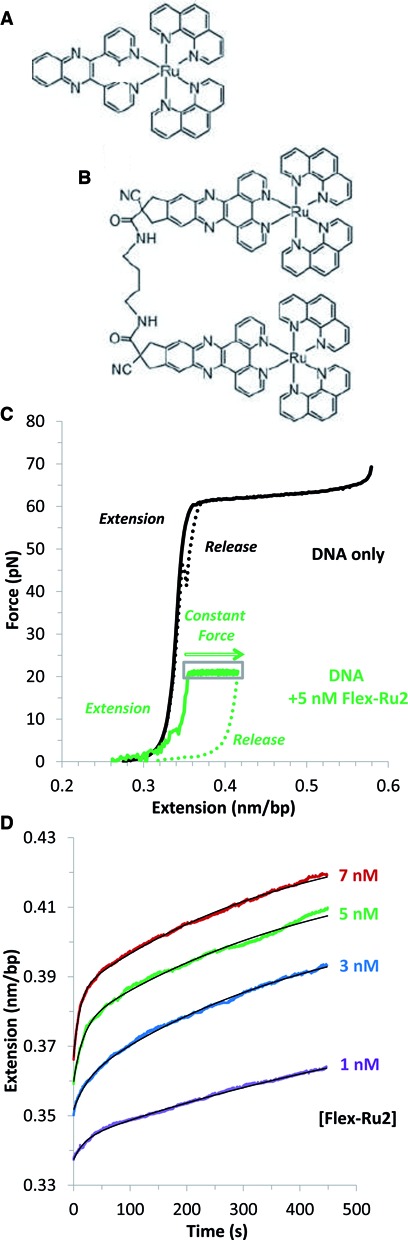
Ru-phen-dppz motifs elongate DNA. (**A**) The large aromatic dipyridophenazine ring of Ru(phen)_2_dppz^2+^ (referred to as Rudppz in the text) intercalates into dsDNA. (**B**) Two Rudppz connected by a flexible linker (the complex referred to as flex-Ru2). (**C**) Cycles of force extension and release for DNA (black lines) and DNA in the presence of 5 nM of flex-Ru2 (green lines). To elucidate the kinetics of flex-Ru2, the force was fixed at 50 pN, 30 pN and 20 pN (20 pN is shown here in the gray box), while the increasing extension was recorded. (**D**) Flex-Ru2 intercalation kinetics for increasing ligand concentrations of 1, 3, 5 and 7 nM (purple, blue, green and red) when held at a force of 20 pN. Fits (black) are to the model of Equation (3), and these results are included in Figures 3 and 4.

Rational drug design is an essential goal for cancer therapy (1), and the flexible ruthenium dimer complex examined here (Figure 1) is part of a series of molecules designed, with that goal in mind, to have a high affinity for DNA and a low dissociation rate. Ruthenium complexes are designed to be DNA intercalators, binding in between DNA base pair (bp) stacks (2–4). This type of binding helps to keep the two strands of a DNA molecule together, which may inhibit cellular replication. Since ruthenium intercalator complexes were first introduced in 1984 (2), significant progress has been made to increase their potential value as cancer therapy or other DNA-targeting drugs. For example, Ru(phen)_3_^2+^ has a binding affinity that is three orders of magnitude greater than Ru(bpy)_3_^2+^ (4). Adding a dppz moiety in place of one of the phen moieties on the Ru(phen)_3_^2+^ molecule results in a binding affinity that is two orders of magnitude greater than the Ru(phen)_3_^2+^ alone (4). Furthermore, linking two Ru(phen)_2_dppz^2+^ (Figure 1A) molecules with a single bond (referred to subsequently as rigid-Ru2 for simplicity) results in binding that requires the molecule to thread one of the bulky Ru(phen)_2_ regions through the base pairs (5–7). This results in very slow association and dissociation kinetics such that the DNA-ruthenium complex does not reach equilibrium on the timescales of typical biochemical and biophysical measurements (6,8,9). In this work we probe the bis-intercalation mechanism of another interesting variant of the Ru(phen)_2_dppz^2+^-(Rudppz)-based molecule, [μ-C4(cpdppz)_2_(phen)_4_Ru_2_]^4+^, which involves two Rudppz groups connected by a flexible four-carbon linker, and we will refer to this molecule as flex-Ru2 (Figure 1B). Here we examine only the (Δ,Δ) isomer for simplicity. The flex-Ru2 molecule was created by analogy to natural antibiotics that bind to DNA by bis-intercalation and achieve high affinity for DNA by linking two or more subunits of known DNA mono-intercalators to form poly-intercalating compounds (10,11). Intercalation of these ligands was traditionally studied by optical spectroscopic approaches, based on the observations that the luminescence of the chiral aromatic groups of these molecules increases greatly upon their intercalation between DNA bases (12). Fine details about positioning of the aromatic groups within the DNA duplex, extent of stacking, as well as overall DNA saturation with the ligand and its kinetics of association and dissociation were addressed (6–8,12–15). However, the timescales for binding of threading intercalators often exceed tens of hours, and therefore the binding mechanisms cannot be adequately studied by these approaches.

In this work we use single molecule DNA stretching in the presence of intercalating ligand to fully characterize equilibrium binding and binding kinetics of flex-Ru2. This method was previously shown to be very informative for studies of ligand-DNA intercalation, as it allows following the intercalation process as a length increase of the ligand-DNA complex (4,5,16–18). Moreover, force exponentially strengthens the equilibrium ligand intercalation by stabilizing the longer DNA state. This was first illustrated in our studies of traditional ‘fast’ intercalators, such as ethidium and mono-intercalating ruthenium-based ligands (including Ru(phen)_2_dppz^2+^) that equilibrate their intercalation on the timescale of our DNA stretching experiment of 10–100 s (3,4,19). For slower intercalators, such as rigid-Ru2 (20) or Actinomycin D (18), the ligand-DNA stretching curves are non-equilibrium, and can be used to study the kinetics of intercalation. This can be done in a variety of ways, from DNA stretching at different rates, to following the kinetics of the DNA length changes upon ligand association or dissociation at a fixed force. The latter approach allows characterization of the ligand-DNA on/off rates as a function of force. As the force typically strongly facilitates the forward intercalation rate, the binding process completes on our observation timescales. The quantitative effect of force on the intercalator on/off rates is determined by the length change of the ligand-DNA complex between the initial state and the rate-limiting transition state, and reports on the molecular mechanism of threading. Extrapolation of the force dependence to zero force allows estimation of the force-free on and off rates, and provides a kinetic estimate of the equilibrium ligand dissociation constant at zero force. Alternatively, equilibrium binding of these slow intercalators can be characterized from the final extension of the ligand-DNA complex after the binding relaxation at a fixed force and for a given ligand concentration. This approach was used to characterize the kinetics and equilibrium of Actinomycin D (18), and was later adapted by others to probe additional complex intercalating ligands (16).

In the present work we adapt our previously developed approach to characterization of DNA bis-intercalation by the flex-Ru2 ligand, which unlike all of the ligands previously studied by DNA stretching occurs in more than one step. Previous optical studies have shown that its two aromatic dppz moieties both intercalate completely and almost identically in between the bp stacks two bp apart from each other, as defined by the flexible 0.75 nm-long four carbon linker threaded through the duplex, and connecting the two dppz moieties on the side of the duplex opposite to the ruthenium phen groups (Figure 1B). Both association and dissociation processes of the flex-Ru2 intercalation appear to be relatively slow and multi-rate. The possibility of kinetically separating mono- or bis- intercalated states for flex-Ru2 was considered but not explored. Instead, the fast and the slow components of the on and off rates of the flex-Ru2 were attributed to heterogeneity of different binding geometries depending on sequence context (21).

We find that the fast and the slow intercalation modes of flex-Ru2 come from the first and the second intercalation events during single ligand molecule DNA binding (Figure 1C). By measuring the ligand concentration dependence of the fast and slow binding rates at several forces and fitting this dependence to a two-step binding model, we show that the fast mode is a bimolecular intercalation of the first dppz moiety, in pre-equilibrium to the ∼10-fold slower and stronger intercalation of the second dppz moiety. We characterize the force-dependence of the binding kinetics and distances to the transition state from each conformation. We estimate the zero-force binding kinetics and equilibrium binding constants for each of the two intercalation steps as well as that of the complete binding process by extrapolating our measured force dependence of these parameters to the force-free state. We conclude that at zero force the flex-Ru2 binding mechanism involves fast initial intercalation by one moiety, followed by a slow conversion to the final bis-intercalated state, and still slower (∼600 s) reverse intercalation of the second moiety. This results in an overall binding affinity that is ∼100-fold greater than that observed for the mononuclear-Ru(phen)_2_dppz^2+^ molecule and also higher than that observed for other threading intercalators, including rigid-Ru2. The extension of our single molecule DNA stretching approach to be able to follow multi-rate intercalation kinetics will be useful for subsequent studies of DNA binding by many intercalating ligands and proteins.

## MATERIALS AND METHODS

### Optical tweezers

The optical tweezers instrument has been previously discussed in greater detail (22,23). A liquid flow cell is placed on a piezoelectric stage between two microscope objectives, while two laser beams are brought to a focus inside the flow cell. The focused beams form the optical trap, which acts as a potential well, trapping a polystyrene microbead coated with streptavidin. Another bead is attached to a micropipette tip. A biotinylated DNA molecule is tethered between these beads and the solution is rinsed with HEPES buffer (100 mM Na^+^, pH 7.5) in preparation for a DNA stretching curve control experiment. DNA stretching behavior has been studied extensively (24–26). Starting at near-zero forces and a low extension, DNA is stretched at a rate of 100 nm/s. As the extension of the molecule increases, it stretches in what is known as the entropic region of the force-extension curve, reaches its contour length of 0.34 nm/bp at 30 pN, and undergoes a phase transition at 62.6 pN (27). These known values for a DNA stretching curve are used to calibrate the force measurements, and to set the initial position of the experiment (28). Once the control force-extension curve is taken, nothing is changed to ensure accurate calibration of the experiments in the presence of ligand.

### Ru-DNA complex experiments

The trapped DNA is returned to a low force and extension; while the ligand solution is flowed (Tris buffer 100 mM Na^+^, pH 8.0, various concentrations of flex-Ru2). Once the DNA molecule was fully in the presence of a solution of uniform ligand concentration, the DNA was rapidly extended (10000 nm/s) to the desired force. Once reached, this target force was maintained with rapid feedback to the piezoelectric stage. The applied force strongly inhibits DNA–DNA contacts that may facilitate DNA-ligand crosslinking. All DNA stretching curves where done in 100 mM Hepes buffer, pH 7.5, and all experiments in the presence of ligand were done in Tris buffer, 100 mM Na+, pH 8.0 at 20°C. The synthesis of the ruthenium complex is described elsewhere (29).

## RESULTS

### Quantifying flex-Ru2/DNA binding from force-extension curves

Presented in Figure 1C are stretch and release curves for DNA alone, which shows a region of entropic elasticity up to 0.34nm/bp B-form DNA contour length, followed by the overstretching transition at ∼62 pN (in 100 mM Na^+^). A solid green line shows the DNA extension curve in the presence of 5 nM flex-Ru2, which shows an increase in length relative to DNA at forces above 5 pN. When the extension curve reaches 20 pN, we initiate a force-feedback loop that increases the DNA length to keep the force constant as the flex-Ru2/DNA complex increases in length when more intercalators bind. Figure 1D shows the extension versus time at a constant force of 20 pN for different forces. The results suggest continuous binding on a timescale of hundreds of seconds. From the fits to the data described below, we obtain the intercalation rates as well as the equilibrium fractional binding for a given force and ligand concentration. Because of the slow ligand dissociation, the dotted return curve reflects the amount of ligand bound at 20 pN, which does not significantly change on the timescale of the release.

### Saturated flex-Ru2/DNA stretching curve reveals DNA interactions of both flex-Ru2 dppz moieties

**Figure 2.**
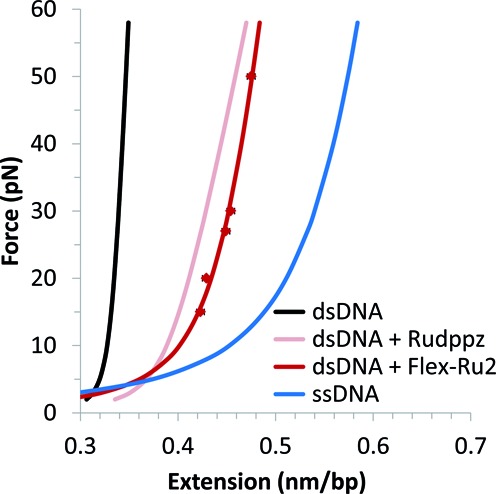
DNA saturated with flex-Ru2 (red), from constant force measurements, and fit to Equation (1). Lines for dsDNA (black) taken from Wenner et al. (27) for dsDNA saturated with Rudppz (pink) from Vladescu et al. (4) and fitted data for ssDNA shown (blue). The parameters of the corresponding WLC fits are collected in Table 1.

Higher concentrations of flex-Ru2 lead to faster ligand association, and to longer equilibrium lengths (Figure 1D). The flex-Ru2 saturated DNA stretching curve, *x_sat_*(*F*), is presented in Figure 2. The five data points that form this curve were obtained as the final equilibrium extensions of flex-Ru2/DNA complex after a 10 min relaxation process performed at constant force in the presence of 20 nM ligand. Also shown in Figure 2 for comparison are the ligand-free single-stranded (ss) and double-stranded (ds) DNA stretching curves, as well as the saturated mono-Rudppz, all fitted to the extensible worm-like chain (WLC) model of polymer elasticity (23) in the form:

(1) \begin{equation*} x_{sat} (F) = x_{sat}^{\max } \cdot \left( {1 - \frac{1}{{2\sqrt {F \cdot A/k_B T} }} + \frac{F}{S}} \right). \end{equation*}

Fitting parameters, $x_{sat}^{\max }$, *A* and *S*, for all four curves are collected in Table 1. The zero-force contour length of the saturated flex-Ru2/DNA complex $x_{sat}^{\max }$ = 0.49 nm/bp is 0.15 nm/bp longer than the contour length of B-form DNA, which is *x_ds_* = 0.34 nm/bp. Using information from previous bulk studies (29) we can assume that the two dppz moieties of each flex-Ru2 ligand intercalate similarly, and that at saturation each ligand occupies a binding site of 4 bp, i.e. every other DNA base stack becomes intercalated. This suggests that the elongation of the complex associated with intercalation of each dppz moiety must be 0.3 nm. For comparison, the saturated zero-force DNA intercalation by mono Rudppz (see pink Rudppz-saturated curve in Figure 2 and the corresponding fit parameters in Table 1) leads to mono-intercalation of every 4 bp at zero force, and its binding site size can be further reduced to ∼3 bp by a high stretching force of ∼50 pN (4). Thus, the compact bis-intercalation of every other stack by the flex-Ru2 dimer leads to a much higher maximum intercalated density of this ligand that is similar to saturated intercalation by the classical intercalator ethidium (4,19). In addition to a longer contour length, the saturated flex-Ru2-DNA complex has a much shorter persistence length (*A*∼3 nm) than either B-form DNA (50 nm) or the saturated Rudppz-DNA complex (15 nm). This shorter persistence length of flex-Ru2-saturated DNA is consistent with frequent intercalation of this ligand, inducing additional random bends in DNA upon binding. Finally, the saturated flex-Ru2 complex has an elastic modulus of ∼800 pN, which is ∼3-fold higher than the elastic modulus of the saturated mono-Ru intercalated DNA, but is 1.5-fold smaller than the elastic modulus of B DNA (Table 1). This high resistance of flex-Ru-saturated DNA to extension beyond its contour length is consistent with a ‘stapling’ effect of flex-Ru2 bis-intercalation, in which every two bp are ‘stapled’ by a short stretched linker (29), which prohibits any further intercalation, as discussed below. In what follows we will use this flex-Ru2-DNA saturated curve *x_sat_*(*F*) to obtain the fractional DNA saturation with this ligand, Θ(*C*,*F*,*t*),for a length of the flex-Ru2-DNA complex, x(*C*,*F*,*t*), as:

(2) \begin{equation*} \Theta (C,F,t) = \frac{{x(C,F,t) - x_{ds} (F)}}{{x_{sat} (F) - x_{ds} (F)}} \end{equation*}

**Table 1.** Comparisons of polymer properties for dsDNA and ssDNA, including dsDNA in saturating concentrations of mono Ru intercalator (Ru1) and flex-Ru2. All parameters determined from fits to Equation (1), which are shown in Figure 2.

Polymer	$x_{sat}^{\max }$ = x_ds_^.^(1+*γ*_0_) (nm/bp)	A (nm)	S (pN)
dsDNA^a^	0.340 ± 0.001	47 ± 2	1270 ± 200
dsDNA + Ru1^b^	0.41 ± 0.01	14.3 ± 0.8	320 ± 17
dsDNA + flex-Ru2^c^	0.49 ± 0.01	2.9 ± 0.8	800 ± 50
ssDNA^d^	0.680 ± 0.002	1.2 ± 0.1	1220 ± 70

^a^Data from Wenner et al. (27).

^b^Data from Vladescu et al. (4).

^c^From this work.

^d^Single-stranded DNA (34 kbps length) in the same solution conditions as the dsDNA in this work.

Values and uncertainties were determined from the minimization of χ^2^ method.

### Constant force experiments characterize the equilibrium and kinetic properties of flex-Ru2-DNA intercalation

The solid black lines in Figure 1D represent fits to the DNA extension as a function of time as the DNA is bound by flex-Ru2 at a constant force of 20 pN. These curves do not fit to a single exponential dependence on time, indicating that the flex-Ru2-DNA binding process is multi-state, unlike the single state process observed for other bis-intercalators (16). A typical elongation versus time *dx(t)* trace can be satisfactory fitted to the two-exponential expression:

(3) \begin{equation*} dx(t) = dx_{eq} - dx_f \cdot e^{ - k_f t} - dx_s \cdot e^{ - k_s t} \end{equation*}

**Figure 3.**
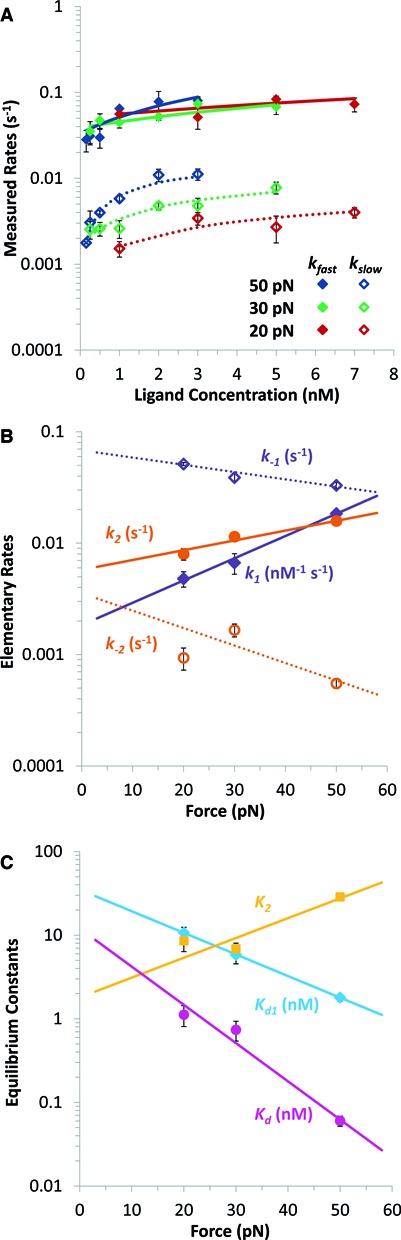
Kinetics of flex-Ru2 binding to dsDNA. (**A**) Measured fast and slow rates versus concentration of flex-Ru2 at three forces: 20, 30 and 50 pN (red, green and blue). Data points are rates *k_f_* and *k_s_* obtained by fitting the length versus time for the flex-Ru2/DNA complex to Equation (3). Lines are the results of fits to Equation (6) (solid lines) and Equation (7) (dotted lines) that determine the elementary rates of the two-step reaction. (**B**) Fitted values of elementary rates of the two-step intercalation, giving the forward rates *k_1_* and *k_2_* (solid purple and orange symbols) and reverse rates *k_−1_* and *k_−2_* (open purple and orange symbols). Lines represent fits to Equation (8), and give the force independent elementary rates and transition distances, as described in the text. Fitted parameters are shown in Table 2. (**C**) Force dependent binding constants for each step *K_d1_* (cyan) and *K_2_* (gold) and for overall binding *K_d_* (magenta), determined from the elementary rates. Lines denote fits to Equation (10), which give the force independent binding constants and equilibrium length changes, which are included in Table 2.

**Figure 4.**
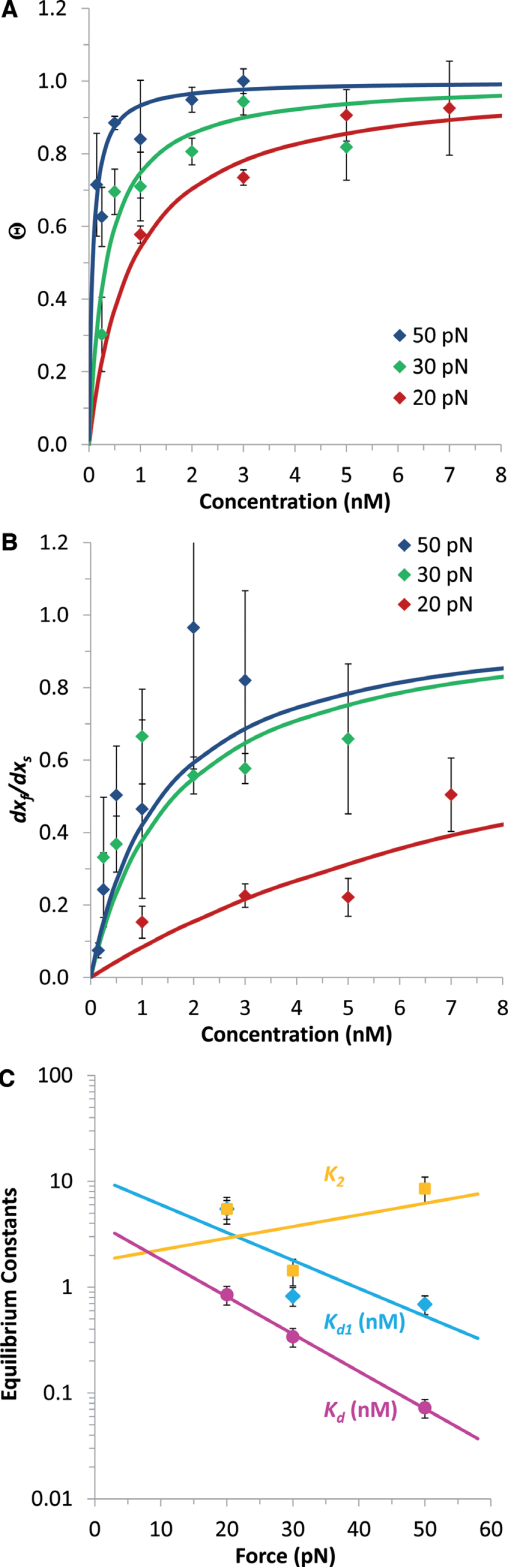
Equilibrium analysis of DNA elongations induced by flex-Ru2 intercalation. (**A**) Measured equilibrium flex-Ru2/DNA length expressed as an occupancy (Θ, relative to the saturated values of Figure 2) as a function of ligand concentration (*C*) for the forces of 20, 30 and 50 pN (red, green and blue). Fits to Equation (12) (lines) determine the binding constant *K_d_* for each force. (**B**) The ratio of the fast and slow elongation amplitudes, *dx_f_*/*dx_s_*, as a function of *C* (20 pN: red, 30 pN: green and 50 pN: blue). Fits of these data points to Equation (15) (lines) determine *K_d1_*(*F*). (**C**) Binding constants *K_d1_* (cyan), *K_d_* (magenta) and *K_2_* (gold) versus force, as obtained from the fits of the data in Figure 4A and B to Equation (10) (lines), with *K_2_* calculated as *K_2_* = *K_d1_*/*K_d_*. Fitted zero-force binding constants and the flex-Ru2/DNA length changes associated with each *K* are collected in Table 2.

Here the fitting is performed for extensions beyond B-DNA contour length, i.e. for *dx*(*t*) *= x*(*t*) *− x_ds._ dx_eq_*(*C,F*) is the equilibrium extension reached by the flex-Ru2/DNA complex, *dx_f_* and *dx_s_* are the amplitudes of the extension changes associated with the fast and the slow binding modes, and *k_f_* and *k_s_* are the rates of the fast and the slow modes, respectively. Because the ligand does not always dissociate on the timescales of these experiments (see Figure 1C), a new DNA molecule is used for each new length relaxation experiment. The constant-force length relaxation is repeated at least three times for each ligand concentration and at three forces (20, 30 and 50 pN) to fully understand the flex-Ru2 binding mechanism. Each ligand concentration and force leads to a unique set of fitted rates, *k_f_* and *k_s_*, and extension amplitudes, *dx_f_* and *dx_s_*, presented in Figures 3A and 4A, respectively. Each of these two sets of fitted parameters can be independently used to completely characterize the kinetics and equilibrium binding properties (from *k_f_* and *k_s_*), or equilibrium only properties (from *dx_f_* and *dx_s_)* of the flex-Ru2-DNA complex, as discussed below. The good agreement between the flex-Ru2-DNA equilibrium binding parameters obtained independently from the two complementary data sets validates the self-consistency of our approach.

Based on the pre-existing notion that the flex-Ru2 ligand has two dppz moieties that can sequentially intercalate duplex DNA, we suggest the following two-step intercalation process (30):

(4) \begin{equation*} Ru + DNA \mathop{\rightleftharpoons}^{k_{1}C}_{k_{-1}} (Ru/DNA)_{fast} \mathop{\rightleftharpoons}^{k_{2}}_{k_{-2}} (Ru/DNA)_{slow} \end{equation*}

Here the first step is the initial fast and reversible intercalation of only one dppz moiety, characterized by the bi-molecular on rate *k_1_* and reverse off rate *k_−1_*. The second step is the mono-molecular conversion from the singly-intercalated to bis-intercalated flex-Ru2 binding with the corresponding forward (*k_2_*) and reverse (*k_−2_*) rates.

#### Analysis of the fast and slow binding yields a complete description of 2-step flex-Ru2/DNA intercalation

A mathematical description developed previously (31) relates the two fitted rates *k_f_* and *k_s_* with four elementary rates of the two-step reaction given by Equation (4). Specifically, under the condition of a much faster first step being in pre-equilibrium to the second slower step, i.e. when

(5) \begin{equation*} k_1 C + k_{ - 1} \gg k_2 + k_{ - 2'} \end{equation*}

where the fast and the slow rates *k_f_* and *k_s_* are related to the elementary reaction rates *k_1_, k_−1_, k_2_* and *k_−2_* as follows:

(6) \begin{equation*} k_f = k_1 C + k_{ - 1} \end{equation*}

(7) \begin{equation*} k_s = k_2 \left( {\frac{{k_1 C}}{{k_1 C + k_{ - 1} }}} \right) + k_{ - 2} \end{equation*}

Presented in Figure 3A are the fitted values of *k_f_* and *k_s_* as a function of ligand concentration (*C*) for 20, 30 and 50 pN. The fits of these dependencies to Equations (6) and (7) yield the elementary reaction rates *k_1_, k_−1_, k_2_* and *k_−2_* as a function of force in Figure 3B. The fact that the experimental *k_f_* (*C*) and *k_s_* (*C*) dependencies are well-described by Equations (6) and (7) supports our two-step intercalation model.

Interestingly, both on and off processes for each intercalation step appear to be exponentially force-dependent and can be well-described by the relationships:

(8) \begin{equation*} k_{ \pm 1,2} (F) = k_{ \pm 1,2}^0 \cdot e^{{{F \cdot x_{ \pm 1,2}^\dagger }/{k_B T}}} \end{equation*}

Here $k_{ \pm 1,2}^0$ are the zero-force rates, and $x_{ \pm 1,2}^\dagger $ are the corresponding length changes either from the unbound to the transition state, $x_{ + 1, + 2}^\dagger $, or from the bound to the transition state, $x_{ - 1, - 2}^\dagger $, for each reaction step. The fitted values of $k_{ \pm 1,2}^0$ and $x_{ \pm 1,2}^\dagger $ parameters for each of the two reaction steps are collected in Table 2. Importantly, the elementary rates satisfy the initial condition of Equation (5) for the faster first intercalation step being in pre-equilibrium to its slower conversion into the doubly-intercalated step at all forces. Thus, the zero-force off rate for the mono-intercalation step, $k_{ - 1}^0$ = (6.8 ± 0.4)^.^10^−2^ s^−1^ is ∼10-fold faster than the conversion step into the bis-intercalated state, $k_{ + 2}^0$ = (5.8 ± 1.0)^.^10^−3^ s^−1^, which is then ∼1.6-fold higher than the dissociation rate for the bis-intercalated flex-Ru2, $k_{ - 2}^0$ = (3.6 ± 1.0)^.^10^−3^ s^−1^.

**Table 2.** Zero-force kinetic and equilibrium parameter for flex-Ru2 bis-intercalation of the dsDNA

	Kinetic approach		Equilibrium approach	
	$K_i^0 \;,\;k_i^0$	$x_i^0$ (nm)	$K_i^0 \;,\;k_i^0$	$x_i^0$ (nm)
*K_d_* (nM)	15 ± 6	0.44 ± 0.04	4.1 ± 1.4	0.33 ± 0.04
*K_d1_* (nM)	35 ± 9	0.24 ± 0.02	11 ± 4	0.25 ± 0.04
*K_2_* (-)	1.8 ± 0.6	0.22 ± 0.03	2.7 ± 0.8	0.08 ± 0.05
*k_1_* (×10^−3^ nM^−1^·s^−1^)	1.8 ± 0.4	0.19 ± 0.02	-	-
*k_−1_* (×10^−3^ s^−1^)	68 ± 4	−0.06 ± 0.01	-	-
*k_2_* (×10^−3^ s^−1^)	5.8 ± 1.0	0.08 ± 0.01	-	-
*k_−2_* (×10^−3^ s^−1^)	3.6 ± 1.0	−0.15 ± 0.03	-	-

The data and the analysis method for the ‘kinetic’ and ‘equilibrium’ approaches are discussed in the main text, and graphically presented in Figures 3 and 4, respectively. Uncertainties determined as errors in the fit directly from each fitting step.

The stretching force facilitates the on rates for both the first and the second intercalation events with corresponding elongations of $x_{ + 1}^\dagger $ = 0.19 ± 0.02 nm and $x_{ + 2}^\dagger $ = 0.08 ± 0.01 nm. Interestingly, the reverse intercalation processes are slowed down by force, and according to Equation (8) are associated with small negative elongations of $x_{ - 1}^\dagger $ = −0.06 ± 0.01 nm and $x_{ - 2}^\dagger $ = −0.15 ± 0.03 nm, implying that the DNA in the transition state is longer than the non-intercalated state, but slightly shorter than in the mono-intercalated state. In contrast, the second transition state is closer to the mono- than to the double-intercalated state. The physical meaning of these fitted kinetic parameter values and their relationship to the structure and intercalation mechanism of the flex-Ru2/DNA complex are considered in the Discussion.

#### Determining the equilibrium binding constants for each intercalation step as well as overall binding

The elementary reaction rates *k_1_, k_−1_, k_2_* and *k_−2_* obtained above can be used to calculate the equilibrium constants for each step and for the net reaction as follows:

(9) \begin{eqnarray*} &&K_{d1} = \frac{{k_{ - 1} }}{{k_1 }} = \frac{C}{{K_1 }},\quad K_2 = \frac{{k_2 }}{{k_{ - 2} }}\quad {\rm and} \nonumber \\ &&K_d = \frac{{K_{d1} }}{{K_2 }} = \frac{{k_{ - 1} }}{{k_1 }} \cdot \frac{{k_{ - 2} }}{{k_2 }} \end{eqnarray*}

Here the *K_d1_ and K_d_* are the dissociation constants for the first step and for the entire reaction, and *K_2_* is the equilibrium constant for the second step. Their values calculated according to Equation (9) are presented as a function of force in Figure 3C. The force dependence for these equilibrium constants are well-fitted by the exponential expressions:

(10) \begin{equation*} K_i (F) = K_i^0 \cdot e^{ - {{F \cdot x_i^0 }/{k_B T}}} \end{equation*}

where $K_i^0$ is the zero-force value of the corresponding equilibrium binding parameter. The zero-force values $K_{d1}^0$ = 35 ± 9 nM, $K_2^0$ = 1.8 ± 0.6 and $K_d^0$ = 15 ± 6 nM demonstrate a high affinity first intercalation event, followed by a strongly driven second intercalation step that makes the overall binding even stronger.

Also, according to the definition of *K_i_*(*F*) given by Equation (9) and expressions for the rates (Equation 8), the equilibrium DNA length change upon intercalation of a single flex-Ru2 molecule in the corresponding binding step (first, second) $x_i^0$ can be found either from fitting of *K_i_*(*F*) to Equation (10), where *K_i_*(*F*) is calculated according to Equation (9) using the elementary reaction rates, or simply from the fitted elongations associated with each elementary reaction rate (see Table 2) as follows:

(11) \begin{equation*} x_i = x_{ + i}^\dagger - x_{ - i}^\dagger \end{equation*}

The first mono-intercalation event leads to flex-Ru2/DNA complex elongation by *x_1_* = 0.19 − (−0.06) = 0.25 nm, and the second intercalation event of the same flex-Ru2 molecule leads to the additional complex elongation by *x_2_* = 0.08 − (−0.15) = 0.23 nm. The total elongation of the DNA-flex-Ru2 complex upon double-intercalation of both dppz moieties of a single ligand molecule is: *x* = *x_1_* + *x_2_* = 0.25 + 0.23 = 0.48 nm. Both of these elongations upon individual intercalation events are shorter than the 0.34 nm elongation predicted by the optical studies (21), and also shorter than the 0.30 nm elongation associated with each intercalation that follows from our fitted contour length of the saturated flex-Ru2/DNA complex (see Table 1). This may be due to the limited accuracy of our fitted rates at very long timescales, which should be especially important for fitting the slow rate values at low forces. Overestimation of the low-force rates would lead to smaller apparent slopes of the ln*(k)* versus *F* dependencies, and therefore, to the lower apparent elongations associated with each process.

#### Equilibrium binding parameters for two-step flex-Ru2-DNA intercalation

Also presented in Table 2 are the same equilibrium flex-Ru2/DNA binding parameters for both steps determined from the fitted equilibrium complex extensions, *dx_f_, dx_s_* and *dx_eq_*. In fitting the flex-Ru2-DNA per base pair extension over time, *dx*(*t*), to Equation (3) we obtain not just the fast and the slow rates of this process, but also the amplitudes of the equilibrium fast, slow and net extension changes, *dx_f_, dx_s_* and *dx_eq_*, all as a function of *C* and *F*. There is wealth of information regarding the equilibrium amounts of each intercalated species in these data that we can use to determine the equilibrium binding characteristics for each step. The total equilibrium extension *dx_eq_* is determined only by the net reaction *K_d_*:

(12) \begin{equation*} dx_{eq} (C,F){=} dx_{sat} (F) \cdot \Theta (C,F) {=} dx_{sat} (F) \cdot \frac{{C/ {K_d }}}{{C/ {K_d }}+1}, \end{equation*}

assuming a simple binding isotherm, while *dx_f_, dx_s_* depend on both *K_d_* and *K_d1_* as follows:

(13) \begin{equation*} dx_f (C,F) {=} dx_{eq} \cdot \frac{f}{2} {=} dx_{sat} (F) \cdot \frac{1}{2}\frac{{C/ {K_d }}}{{C/ {K_d }}+1}\cdot \frac{{C/ {K_{d1} }}}{{C/ {K_{d1} }}+1} \end{equation*}

and

(14) \begin{eqnarray*} &&dx_s (C,F) = \\
&&dx_{eq} \left( {1 - \frac{f}{2}} \right) = dx_{sat} (F) \cdot \frac{{C/ {K_d }}}{{C/ {K_d }}+1}\left ( 1-\frac{1}{2} \frac{{C/ {K_{d1} }}}{{C/ {K_{d1}+1 }}} \right ), \end{eqnarray*}

where $f = \frac{{C/ K_{d1} }}{{C/ K_{d1}+1}}$ is the equilibrium probability of the mono-intercalated state. However, the *dx_f_*/*dx_s_* ratio is only determined by *K_d1_*:

(15) \begin{equation*} \frac{{dx_f }}{{dx_s }} = \frac{f/2}{1-f/2} = \frac{C/K_{d1}}{C/K_{d1}+2} \end{equation*}

The factor ${f/2}$ in Equations (13)–(15) appears as a result of the assumption that the DNA-flex-Ru2 complex becomes longer by the same amount during the first and second intercalation events. Presented in Figure 4A and B are the normalized value of the equilibrium extension $\Theta (C,F) = dx_{eq}(C,F)/dx_{sat}(F)$ (calculated according to Equation 2), and the ratio of the fast and slow equilibrium extensions *dx_f_*/*dx_s_*, as a function of *C* for three different *F* values. The experimental data points are fitted to Equations (12) and (15) yielding *K_d_* and *K_d1_*, respectively. These *K_d_* and *K_d1_* values, along with *K_2_ = K_d1_/K_d_* for the three forces studied, are presented in Figure 4C. These force dependencies of *K_d_, K_d1_* and *K_2_* are further fitted to Equation (10), yielding the zero-force values of the equilibrium dissociation and binding constants, as well as their corresponding changes in the length of the flex-Ru2/DNA complex. The values of these parameters determined with this equilibrium data analysis method collected in Table 2 are not identical to, but semi-quantitatively consistent with, the kinetic estimates of the same parameters described above, which are also presented in Table 2. In both cases, the zero-force net dissociation constant for the flex-Ru2/DNA bis-intercalation *K_d_* is about half the value for just the first mono-intercalation step, *K_d1_*, due to the second intercalation step being strongly driven, as follows from its equilibrium constant of *K_2_* ∼2. In both cases the net bis-intercalation is quite strong with *K_d_* in the 1–10 nM range. In addition, the magnitudes of DNA extension due to flex-Ru2 mono and bis-intercalation are only slightly smaller when determined from the equilibrium analysis, compared to the values obtained from kinetic analysis. As our kinetic data (the fitted flex-Ru2/DNA length relaxation rates) and the equilibrium data (the magnitudes of fast, slow and total equilibrium extensions, see Equation 3) contain independent information, these two approaches are complementary. The good agreement between the results of these two approaches strongly supports the conclusions of this study.

It is important to mention here that our kinetic approach is more reliable than the equilibrium one. This is because the rates are universal, and do not depend on the initial and the final extensions of the system. Furthermore, the slow and the total extension amplitude accuracies are limited by the positional drift of the optical tweezers instrument over the long timescales of threading intercalation. In addition, the fast amplitude accuracy may also be affected by ligand binding before the initial stretch (although this should be small due to low binding affinity at zero force) as well as limitations in instrument speed at the fastest rates. These factors lead to additional uncertainty in the amplitudes, which is reflected in the higher uncertainties for the data acquired from these amplitude measurements.

## DISCUSSION

In this work we find that the saturated flex-Ru2/DNA complex is 44% longer than B-form DNA, consistent with each of the two dppz moieties of flex-Ru2 being fully intercalated, extending the duplex by ∼0.30 nm each at every other bp stack (Figure 2 and Table 1). This result is consistent with previous measurements of the flex-Ru2/DNA binding stoichiometry by optical methods (21), which show that the binding site size of flex-Ru2 is 4 bp per molecule, or 2 bp per dppz moiety, with similar stacking of each set of dppz aromatic rings with DNA. We also find that the saturated flex-Ru2-DNA complex is ∼16-fold more flexible than B-form DNA. Indeed, the persistence length of the saturated flex-Ru2-DNA complex is ∼3 nm and this length contains ∼6 bp of saturated flex-Ru2/DNA complex. This persistence length of only 6 bp is just a bit larger than the 4 bp binding site size of flex-Ru2. Therefore, the saturated flex-Ru2/DNA complex behaves as a polymer with a free random bend at almost every ligand binding site. Also, the saturated flex-Ru2/DNA complex is ∼1.5-fold more extensible than B-form DNA (elastic modulus of ∼800 pN), but ∼3-fold less extensible than the saturated mono-Rudppz intercalated DNA (see Table 1), consistent with a fairly inextensible DNA defined by the ‘stapling’ of every other adjacent bp by the 0.75 nm four-carbon linker, as previously suggested (21,29).

**Figure 5.**
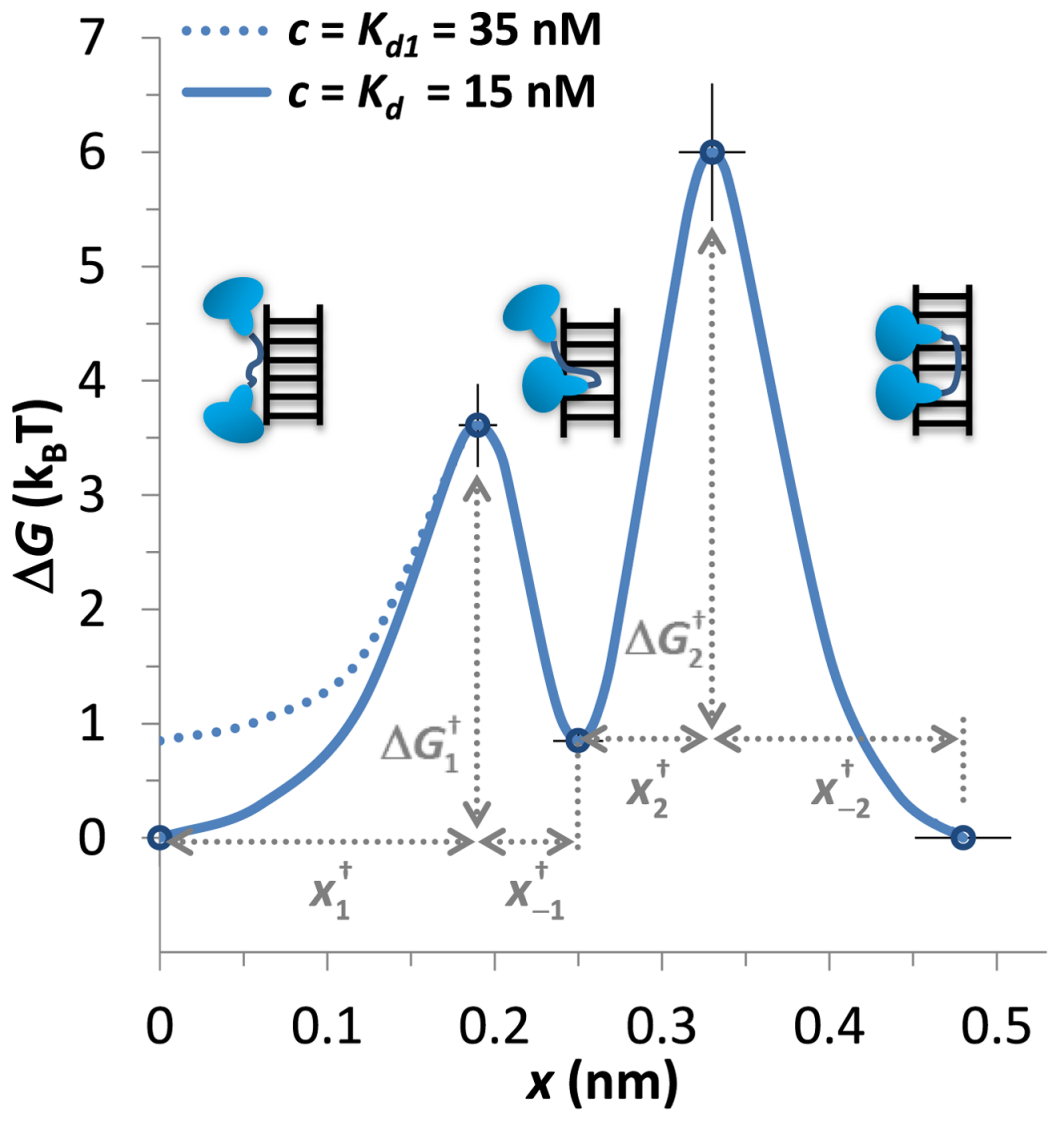
Zero-force free energy profile of the DNA/flex-Ru2 complex versus elongation. The three free-energy minima on this diagram correspond to the non-intercalated, mono-intercalated and bis-intercalated states illustrated in the figure. At the flex-Ru2 concentration of *C = K_d_ =* 15 nM, the free energy of the non-intercalated and bis-intercalated states are the same and are taken here as the zero free energy reference state. At higher *C = K_d1_* = 35 nM the free energies of the non-intercalated and the mono-intercalated states are the same, and equal to *k_B_T*^.^ln(*K_2_*) = *k_B_T*^.^ln(35/15) = 0.85 *k_B_T*. Only the non-intercalated state free energy is affected by *C*, as illustrated by the two lines (solid line for 15 nM and dashed line for 35 nM). The free energy barriers $\Delta G_{1,2}^\dagger $ between the local free energy minima were calculated, as discussed in the main text, and all extensions are taken from Table 2.

The kinetics of flex-Ru2/DNA binding can be minimally described as bi-exponential (Figure 3), and is consistent with a two-step sequential intercalation of two dppz moieties of this ligand (Equation 1), in contrast to previously measured bis-intercalators (16,32,33). Our measured force-dependencies for the four elementary reaction rates (Figure 3A) of this process yield the zero-force values of all rates, and the elongation of the flex-Ru2/DNA complex associated with each of these four processes, as summarized in Table 2. This information is also presented graphically in Figure 5 as a zero-force free energy profile of the DNA/flex-Ru2 complex as a function of its elongation. The three free-energy minima on this diagram correspond to the non-intercalated, mono-intercalated and bis-intercalated states. At the solution concentration of flex-Ru2 of *C = K_d_ =* 15 nM the free energy of the non-intercalated and bis-intercalated states are the same, and are taken here as a zero free energy reference state. At higher *C = K_d1_* = 35 nM the free energies of the non-intercalated state and of the mono-intercalated states are the same, and equal to *k_B_T*^.^ln(*K_2_*) = *k_B_T*^.^ln(35/15) = 0.85 *k_B_T* relative to the reference state. Only the non-intercalated state free energy is affected by *C*, as illustrated in Figure 5 by its two free energy values 0 and 0.85 *k_B_T*, corresponding to 15 nM and 35 nM of flex-Ru2, respectively. The free energy barriers between the local free energy minima define the elementary rate constants, but can only be estimated up to the unknown constant *k_B_T*^.^ln(*k_0_*), as $\Delta G_i^\dagger $ = *k_B_T*^.^ln(*k_0_/*$k_i^0$), where $k_i^0$ are the zero-force rates of the on and off processes for the first or the second step (i.e. *i* = ±1 or ±2), and *k_0_* is the unknown attempt rate. The transition free energies shown in Figure 5 were calculated for *k_0_* = 1 s^−1^. The much higher second barrier $\Delta G_{2}^\dagger $ = − *k_B_T*^.^ln(5.8.10^−3^) = 5.1 *k_B_T* reflects the slower second intercalation transition compared to the first one with $\Delta G_1^\dagger $ (*C* = 15 nM) = *k_B_T*^.^ln(15^.^1.8^.^10^−3^) = 3.6 *k_B_T*.

The extensions of the complex at each free energy minimum and at the transition states are derived from the force dependence of all elementary reactions rates (summarized in Table 2), and are shown in Figure 5 relative to non-intercalated complex length. Despite the limited experimental accuracy of our extension values, the main semi-quantitative conclusions summarized in Figure 5 are quite robust. Thus, for both intercalation steps the transition state is in between the non-intercalated and intercalated states, such that the complex extension increases continuously, as the flex-Ru2 molecule transitions from its non-intercalated to bis-intercalated state. Furthermore, the positions of both transition states are highly asymmetric. Thus, during the first intercalation only the on- process is associated with significant complex lengthening by $x_{ + 1}^\dagger $ = 0.19 nm, leading to the unstable intercalated transition state, which requires only minor additional elongation by −$x_{ - 1}^\dagger $ = 0.06 nm to become a stable mono-intercalated state. The reciprocal off process for the first dppz intercalation thus does not involve major duplex deformation, and is rather fast, with a timescale of ${1/ {k_{ - 1}^0 }}$∼ 15 s. This off process for the mono-intercalated state is clearly not rate-limited by dppz unstacking, which by itself is known to only take ∼1 s (21,29), and would be associated with significant complex shortening. Instead, as the C4 linker is located at the intercalating edge of dppz moiety, we hypothesize that it is the passing of C4 linker through the DNA duplex that rate-limits both on and off processes during the first intercalation step. This hypothesis is consistent with the dissociation timescale for the intercalated mono-Rudppz with the attached C4 (flex-Ru2 without second Ru complex) being ∼15 s (21,29). The fact that the on rate for the first intercalation step in flex-Ru2/DNA binding, *C*^.^*k_1_*, is bi-molecular implies that there is an additional fast and unstable non-intercalative binding mode of flex-Ru2 to DNA that is in pre-equilibrium to the next slower intercalation. Furthermore, the observation that most of the complex elongation that occurs during mono-intercalation is associated with the association process implies that dppz intercalation occurs in rapid pre-equilibrium to the slower process stabilizing this mono-intercalated step. This is an example of a multi-step process for which the kinetics of its faster less stable steps cannot be distinguished, and the net on-rate is modified by the equilibrium constants of these prior steps in rapid pre-equilibrium to the rate-limiting step. This initial process can be characterized by a single high transition barrier.

Once the mono-intercalated state is stabilized, the flex-Ru2/DNA complex is further driven toward bis-intercalation due to *K_2_* = ${k_{+ 2}^0}/{k_{ - 2}^0}$ = 1.8 > 1, resulting in additional lowering of the complex free energy by *k_B_T*^.^ln(*K_2_*) = 0.59 *k_B_T* after the second intercalation event. This second intercalation event is much slower, ${1/{k_2^0 }}$∼ 170 s, and is not rate-limited by intercalation itself, as it is accompanied by a minor complex elongation of just $x_{ + 2}^\dagger $ = 0.08 nm. This slow process must involve a conformational adjustment in the C4 linker, positioning the second dppz moiety in a state optimal for intercalation. This C4 conformational change is by itself highly unfavorable, leading to the second transition barrier, which is unfavorable relative to the mono-intercalated state by 5.63 − 0.85 = 4.8 *k_B_T*. It is then stabilized by a fast second intercalation event leading to significant complex extension by −$x_{ - 2}^\dagger $ = 0.15 nm. The reciprocal slow ${1/{k_{-2}^{0}}}$ = 280 s off process for the second intercalation step has the intercalation itself in fast pre-equilibrium to the subsequent slow conformational change in C4, which rate-limits both on and off processes for the second intercalation. The net DNA elongation upon bis-intercalation of the single flex-Ru2 molecule is 0.48 nm, of which $x_1 = x_{ + 1}^\dagger - x_{ - 1}^\dagger $ = 0.25 nm comes from the first, and $x_2 = x_{ + 2}^\dagger - x_{ - 2}^\dagger $ = 0.23 nm comes from the second intercalated dppz moiety. This result is semi-quantitatively consistent with the conclusion from the WLC analysis of the saturated flex-Ru2/DNA intercalated complex (see Figure 2 and Table 1), suggesting that the double-intercalation of flex-Ru2 leads to DNA extension per ligand of 0.60 nm, or 0.3 nm per dppz intercalation event.

We can now compare our results on flex-Ru2/DNA bis-intercalation to the results of the previous solution studies on flex-Ru2 and related ligands. Previous studies presented models of the doubly-intercalated flex-Ru2/DNA complex obtained by free-energy minimization of the complex using molecular dynamic simulations (21,29). This model was based on information obtained in the optical study (29), suggesting that the flex-Ru2 intercalates B-form DNA with both dppz moieties, each in a similar way, without interaction between the intercalated dppz moieties, and with a binding sites size of 4 bp per flex-Ru2 ligand. In addition, energy minimization of the flex-Ru2/DNA complex suggested that both dppz moieties intercalate from the minor groove side of B DNA and are separated by one non-intercalated base stack. This conformation allows for the optimum stacking of each dppz moiety, and the additional interactions of the Ruphen side groups with the DNA minor groove, and is also consistent with the ∼0.75 nm length of the stretched C4 linker ‘stapling’ two bp stacks on the opposite side of the duplex. This equilibrium state model is fully consistent with our flex-Ru2/DNA stretching results summarized above. In addition, we show that the two dppz moieties intercalate sequentially, one after another, with the second step rate-limited by the slow passage of the C4 linker through the base pairs to allow the two dppz moieties to intercalate in the same orientation and to be separated by only one base stack.

The flex-Ru2 off rates measured in this study can also be compared to the off rates measured by the sodium dodecyl sulphate (SDS) capture assay (21,29). A bi-exponential off process for flex-Ru2 dissociation from calf thymus DNA was observed. The two off rates were measured: *k_off1_* = 6.3^.^10^−3^ s^−1^ = 1/(160 s) and *k_off2_* = 10^−3^ s^−1^ = 1/(1000 s) in 100 mM NaCl. These two rates were attributed to the DNA sequence dependence of flex-Ru2 dissociation kinetics, which becomes mono-exponential with only *k_off1_* ∼6^.^10^−3^ s^−1^ = 1/(170s) for flex-Ru2 dissociation from poly(dA-dT) DNA. However, in their subsequent work the authors have shown that the SDS capture method to significantly overestimates the intercalation off rates (9). Therefore, the faster of the two SDS capture-measured rates ∼1/(170 s), most likely, corresponds to the slowest off rate $k_{ - 2}^0$ = 1/(280 s) in our experiments, which is enhanced by SDS in the previous work. The slower rate *k_off2_* = 1/(1000 s), is most likely beyond our ability to follow completely with DNA stretching, and is fitted with a single slow component during our ∼600 s measurement. Likewise, the long-time measurements during the solution SDS capture are unable to detect the faster off process for mono-dppz unstacking $k_{ - 1}^0$ = 0.068 s^−1^ characterized in the present work. Therefore, the double-exponential flex-Ru2/DNA binding kinetics in our experiments is clearly the result of a two-state intercalation, and is not associated with the DNA sequence dependence of flex-Ru2 binding. We can also confidently dismiss the possibility that the bi-exponential flex-Ru2/DNA intercalation kinetics may come from different DNA interactions of the two possible enantiomeric state of flex-Ru2, as only one flex-Ru2 isoform (Δ, Δ) was used in the present study.

The case of flex-Ru2/DNA bis-intercalation characterized in the present study can be contrasted with the mono-intercalation of the rigid-Ru2 ligand described in our previous work (5,20). The rigid-Ru2 ligand is different from the flex-Ru2 ligand studied here only by the absence of the C4 linker, as the two Rudppz groups are instead connected by a single covalent bond. The rigid-Ru2 intercalates DNA with only one dppz moiety in a single step with a bi-molecular on rate of 10^−5^ nM^−1.^s^−1^, which is 100-fold slower than the first dppz intercalation event for flex-Ru2. Also, the off rate k_off_ ∼1/(700 s) is ∼50-fold slower than the off rate for the first intercalation event of flex-Ru2. Importantly, both on and off processes for rigid-Ru2 intercalation are associated with very large DNA elongations of 0.33 nm and 0.14 nm, respectively. In other words, both on and off processes are strongly facilitated by DNA stretching force. This is in contrast to both flex-Ru2 intercalation events, for which the on rates are facilitated by force but off rates are inhibited by force (see Table 2 and Figure 5). Interestingly, the net DNA elongation upon equilibrium dppz intercalation of rigid-Ru2 is ∼0.29 nm, which is similar to the DNA elongation associated with the first dppz intercalation ∼0.25 nm of flex-Ru2. Thus, it is mostly the ∼100-fold slower kinetics for the rigid-Ru2 that distinguishes the mono-intercalation events for these two Ru2 ligands. The very slow kinetics of rigid-Ru2 intercalation is clearly rate-limited by the slow ‘threading’ of its bulky out of plane Ru-phen groups through the duplex required for both on and off processes. This is in stark contrast to the case of the both flex-Ru2-dppz intercalation events, for which the intercalation events themselves are fast, while the conformational changes in the C4 linker occurring in between the two intercalation events, and stabilizing both of them, are rate-limiting, but are not associated with major duplex lengthening. In the latter case of flex-Ru2 intercalation, only one of the rates (on or off) for each of the two intercalation steps is associated with significant DNA elongation, while the reverse process leads to only minor additional elongations of the complex. In the case of bulkier ‘threading’ intercalation, both on and off processes require major duplex elongations, associated with strong local duplex destabilization. The latter process leads to the ∼100-fold slower kinetics of rigid-Ru2 intercalation relative to the mono-intercalation of flex-Ru2. At the same time, the apparent slow off rate for the flex-Ru2 molecule is defined by the slow conformational changes in this molecule as it binds, with duplex shortening associated with reverse intercalation of the second dppz in fast pre-equilibrium. As a result, the flex-Ru2 molecule combines overall fast association kinetics, $k_{-1}^0=k_1^0$ ∼ 1/(15 s) at *K_d1_* = 35 nM, with slow off kinetics $k_{ - 2}^0$∼1/(280 s) and strong binding, *K_d_* ∼ 15 nM, a combination of qualities suitable for an anticancer drug.

Finally, it is instructive to compare flex-Ru2/DNA intercalation with the intercalation of the single mono-Rudppz molecule characterized in our previous work (4). The mono-intercalation of Rudppz is at least 10-fold faster than even the fastest first step of flex-Ru2 intercalation [k > 1/(15 s)]. However, the Rudppz mono intercalation affinity, with *K_d_* = 1100 nM, is much weaker than either mono- or bis- intercalation of flex-Ru2 with *K_d_* ∼ 35nM and 15 nM, respectively. The much stronger binding of Flex-Ru2 is probably due to the additional non-intercalative electrostatic interactions of flex-Ru2 (4+) with DNA, relative to Rudppz (2+) as well as the additional interactions between the flex-Ru2 phen side groups in the minor groove of DNA. Our ability to characterize such diverse scenarios of DNA intercalation by closely related Ru-based ligands illustrates the power of the single molecule DNA stretching approaches to characterize the intercalation mechanisms of slow ligands in unprecedented quantitative detail. The method developed here can also be applied to other types of ligands and biomolecules that may increase DNA length upon binding in multiple steps.

Regarding flex-Ru2 therapeutic applicability, its higher affinity may help to reduce a potential treatment dose, but other biological factors should be considered for rational drug design. In an *in vitro* study using living non-cancer cells, flex-Ru2 did not inhibit replication due to low membrane penetration (34). Therefore, an effective drug delivery scheme combined with structural ligand modifications is needed to enhance cellular uptake of flex-Ru2 (35,36). The high DNA binding affinity of this ligand is otherwise insufficient to make it a good candidate for an anticancer drug. However, other ruthenium complexes have previously shown cytotoxicity (37,38). Thus it is possible to optimize such complexes based on the threading characteristics of flex-Ru2 in order to synthesize potentially effective anti-cancer drugs. Our study suggests specific mechanisms of DNA interaction that maximize molecular characteristics that are desirable for anti-cancer drugs. In particular, its two-step binding mechanism gives flex-Ru2 (*K_d_*(0) = 15 nM) a higher binding affinity than the rigid-Ru2 molecule (*K_d_*(0) = 44 nM), which contains the same moieties configured to allow only one-step binding. In contrast, the actively used anticancer drug Actinomycin D (39) has on and off rates that are a factor of ten lower than those of flex-Ru2 (18), but also has a one-step binding mechanism with an overall binding affinity (*K_d_*(0) = 1.2 μM) that is much lower than that of flex-Ru2. These results suggest that it may be useful to target the development of multi-step DNA binding ligands similar to flex-Ru2 to optimize DNA binding affinity, but with the slower off rates characteristic of the one-step ligands such as rigid-Ru2 and Actinomycin D. Unlike flex-Ru2, both of these ligands bind DNA dynamically through a lock mechanism, in which DNA length must increase to allow ligand dissociation, to limit their off rates (18,20). Hence it may be desirable to combine two-step binding with a lock mechanism.

## References

[1] Muller W., Crothers D.M. (1968). Studies of the binding of actinomycin and related compounds to DNA. J. Mol. Biol..

[2] Barton J.K., Danishefsky A., Goldberg J. (1984). Tris(phenanthroline)ruthenium(II): stereoselectivity in binding to DNA. J. Am. Chem. Soc..

[3] Mihailovic A., Vladescu I., McCauley M., Ly E., Williams M.C., Spain E.M., Nunez M.E. (2006). Exploring the interaction of ruthenium(II) polypyridyl complexes with DNA using single-molecule techniques. Langmuir.

[4] Vladescu I.D., McCauley M.J., Nunez M.E., Rouzina I., Williams M.C. (2007). Quantifying force-dependent and zero-force DNA intercalation by single-molecule stretching. Nat. Methods.

[5] Paramanathan T., Westerlund F., McCauley M.J., Rouzina I., Lincoln P., Williams M.C. (2008). Mechanically manipulating the DNA threading intercalation rate. J. Am. Chem. Soc..

[6] Wilhelmsson L.M., Westerlund F., Lincoln P., Norden B. (2002). DNA-binding of semirigid binuclear ruthenium complex delta,delta-[mu-(11,11’-bidppz)(phen)(4)ru(2)](4+): extremely slow intercalation kinetics. J. Am. Chem. Soc..

[7] Nordell P., Westerlund F., Wilhelmsson L.M., Norden B., Lincoln P. (2007). Kinetic recognition of AT-rich DNA by ruthenium complexes. Angew. Chem. Int. Ed..

[8] Westerlund F., Nordell P., Norden B., Lincoln P. (2007). Kinetic characterization of an extremely slow DNA binding equilibrium. J. Phys. Chem. B.

[9] Westerlund F., Wilhelmsson L.M., Norden B., Lincoln P. (2003). Micelle-sequestered dissociation of cationic DNA-intercalated drugs: unexpected surfactant-induced rate enhancement. J. Am. Chem. Soc..

[10] Wang A.H.J., Ughetto G., Quigley G.J., Hakoshima T., Vandermarel G.A., Vanboom J.H., Rich A. (1984). The Molecular-Structure of a DNA Triostin-a Complex. Science.

[11] Waring M.J., Wakelin L.P. (1974). Echinomycin: a bifunctional intercalating antibiotic. Nature.

[12] Lincoln P., Norden B. (1996). Binuclear ruthenium(II) phenanthroline compounds with extreme binding affinity for DNA. Chem. Comm..

[13] Nordell P., Lincoln P. (2005). Mechanism of DNA threading intercalation of binuclear Ru complexes: Unior bimolecular pathways depending on ligand structure and binding density. J. Am. Chem. Soc..

[14] Nordell P., Westerlund F., Reymer A., El-Sagheer A.H., Brown T., Norden B., Lincoln P. (2008). DNA polymorphism as an origin of adenine-thymine tract length-dependent threading intercalation rate. J. Am. Chem. Soc..

[15] Westerlund F., Nordell P., Blechinger J., Santos T.M., Norden B., Lincoln P. (2008). Complex DNA binding kinetics resolved-by combined circular dichroism and luminescence analysis. J. Phys. Chem. B.

[16] Biebricher A.S., Heller I., Roijmans R.F.H., Hoekstra T.P., Peterman E.J.G., Wuite G.J.L. (2015). The impact of DNA intercalators on DNA and DNA-processing enzymes elucidated through force-dependent binding kinetics. Nat. Commun..

[17] Camunas-Soler J., Manosas M., Frutos S., Tulla-Puche J., Albericio F., Ritort F. (2015). Single-molecule kinetics and footprinting of DNA bis-intercalation: the paradigmatic case of Thiocoraline. Nucleic Acids Res..

[18] Paramanathan T., Vladescu I., McCauley M.J., Rouzina I., Williams M.C. (2012). Force spectroscopy reveals the DNA structural dynamics that govern the slow binding of Actinomycin D. Nucleic Acids Res..

[19] Vladescu I.D., McCauley M.J., Rouzina I., Williams M.C. (2005). Mapping the phase diagram of single DNA molecule force-induced melting in the presence of ethidium. Phys. Rev. Lett..

[20] Almaqwashi A.A., Paramanathan T., Lincoln P., Rouzina I., Westerlund F., Williams M.C. (2014). Strong DNA deformation required for extremely slow DNA threading intercalation by a binuclear ruthenium complex. Nucleic Acids Res..

[21] Onfelt B., Lincoln P., Norden B. (2001). Enantioselective DNA threading dynamics by phenazine-linked [Ru(phen)(2)dppz](2+) dimers. J. Am. Chem. Soc..

[22] McCauley M.J., Williams M.C. (2009). Optical tweezers experiments resolve distinct modes of DNA-protein binding. Biopolymers.

[23] Chaurasiya K.R., Paramanathan T., McCauley M.J., Williams M.C. (2010). Biophysical characterization of DNA binding from single molecule force measurements. Phys. Life Rev..

[24] Williams M.C., Wenner J.R., Rouzina I., Bloomfield V.A. (2001). Entropy and heat capacity of DNA melting from temperature dependence of single molecule stretching. Biophys. J..

[25] Williams M.C., Wenner J.R., Rouzina I., Bloomfield V.A. (2001). Effect of pH on the overstretching transition of double-stranded DNA: evidence of force-induced DNA melting. Biophys. J..

[26] Williams M.C., Rouzina I., Bloomfield V.A. (2002). Thermodynamics of DNA interactions from single molecule stretching experiments. Acc. Chem. Res..

[27] Wenner J.R., Williams M.C., Rouzina I., Bloomfield V.A. (2002). Salt dependence of the elasticity and overstretching transition of single DNA molecules. Biophys. J..

[28] Rickgauer J.P., Fuller D.N., Smith D.E. (2006). DNA as a metrology standard for length and force measurements with optical tweezers. Biophys. J..

[29] Onfelt B., Lincoln P., Norden B. (1999). A molecular staple for DNA: threading bis-intercalating [Ru(phen)(2)dppz](2+) dimer. J. Am. Chem. Soc..

[30] Chaurasiya K.R., McCauley M.J., Wang W., Qualley D.F., Wu T., Kitamura S., Geertsema H., Chan D.S., Hertz A., Iwatani Y. (2014). Oligomerization transforms human APOBEC3G from an efficient enzyme to a slowly dissociating nucleic acid-binding protein. Nat. Chem..

[31] Vo M.N., Barany G., Rouzina I., Musier-Forsyth K. (2006). Mechanistic studies of mini-TAR RNA/DNA annealing in the absence and presence of HIV-1 nucleocapsid protein. J. Mol. Biol..

[32] Paik D.H., Perkins T.T. (2012). Dynamics and multiple stable binding modes of DNA intercalators revealed by single-molecule force spectroscopy. Angew. Chem. Int. Ed. Engl..

[33] Murade C.U., Subramaniam V., Otto C., Bennink M.L. (2009). Interaction of oxazole yellow dyes with DNA studied with hybrid optical tweezers and fluorescence microscopy. Biophys. J..

[34] Onfelt B., Gostring L., Lincoln P., Norden B., Onfelt A. (2002). Cell studies of the DNA bis-intercalator Delta-Delta [mu-C4(cpdppz)(2)-(phen)(4)Ru(2)](4+): toxic effects and properties as a light emitting DNA probe in V79 Chinese hamster cells. Mutagenesis.

[35] Puckett C.A., Barton J.K. (2009). Fluorescein redirects a ruthenium-octaarginine conjugate to the nucleus. J. Am. Chem. Soc..

[36] Puckett C.A., Barton J.K. (2010). Targeting a ruthenium complex to the nucleus with short peptides. Bioorg. Med. Chem..

[37] Pisani M.J., Weber D.K., Heimann K., Collins J.G., Keene F.R. (2010). Selective mitochondrial accumulation of cytotoxic dinuclear polypyridyl ruthenium(II) complexes. Metallomics.

[38] Chen Y., Qin M.Y., Wang L., Chao H., Ji L.N., Xu A.L. (2013). A ruthenium(II) beta-carboline complex induced p53-mediated apoptosis in cancer cells. Biochimie.

[39] Farber S., D'Angio G., Evans A., Mitus A. (1960). Clinical studies on actinomycin D with special reference to Wilms’ tumor in children. Ann. N. Y. Acad. Sci..

